# Gender Characteristics of the Novel Coronavirus Infection (COVID-19) in Middle-Aged Adults

**DOI:** 10.17691/stm2021.13.4.02

**Published:** 2021-08-28

**Authors:** E.S. Nekaeva, A.E. Bolshakova, E.S. Malysheva, E.A. Galova, E.V. Makarova, T.A. Nekrasova, I.V. Polyakova, Z.S. Bedretdinova, D.V. Belikina, A.A. Lavrenyuk, I.V. Fomin

**Affiliations:** Head of the Admission and Consultation Department, University Clinic; Specialist in Internal Diseases, University Clinic; Privolzhsky Research Medical University, 10/1 Minin and Pozharsky Square, Nizhny Novgorod, 603005, Russia; Specialist in Assessing Pharmacological Prescriptions, Admission and Consultation Department, University Clinic; Associate Professor, Department of Pharmaceutical Chemistry and Pharmacognosy; Privolzhsky Research Medical University, 10/1 Minin and Pozharsky Square, Nizhny Novgorod, 603005, Russia; Assistant, Department of Endocrinology and Internal Medicine; Endocrinologist, Admission and Consultation Department, University Clinic; Privolzhsky Research Medical University, 10/1 Minin and Pozharsky Square, Nizhny Novgorod, 603005, Russia; Deputy Director for Science, University Clinic; Associate Professor, Department of Public Health and Healthcare; Privolzhsky Research Medical University, 10/1 Minin and Pozharsky Square, Nizhny Novgorod, 603005, Russia; Associate Professor, Head of the Department of Propedeutics of Internal Diseases; Deputy Director for Academic Affairs, Institute of Internal Diseases; Privolzhsky Research Medical University, 10/1 Minin and Pozharsky Square, Nizhny Novgorod, 603005, Russia; Associate Professor, Professor, Department of Endocrinology and Internal Medicine; Privolzhsky Research Medical University, 10/1 Minin and Pozharsky Square, Nizhny Novgorod, 603005, Russia; Assistant, Vogralik Department of Advanced Internal Medicine and General Medical Practice; Specialist in Internal Diseases, Consultative and Diagnostic Department with Radiation Diagnostics and Day Hospital, University Clinic; Privolzhsky Research Medical University, 10/1 Minin and Pozharsky Square, Nizhny Novgorod, 603005, Russia; Specialist in Internal Diseases, Consultative and Diagnostic Department with Radiation Diagnostics and Day Hospital, University Clinic; Privolzhsky Research Medical University, 10/1 Minin and Pozharsky Square, Nizhny Novgorod, 603005, Russia; Assistant, Department of Endocrinology and Internal Medicine; Privolzhsky Research Medical University, 10/1 Minin and Pozharsky Square, Nizhny Novgorod, 603005, Russia; Student, Medical Faculty; Privolzhsky Research Medical University, 10/1 Minin and Pozharsky Square, Nizhny Novgorod, 603005, Russia; Professor, Head of the Vogralik Department of Advanced Internal Medicine and General Medical Practice; Director of the Institute of Internal Diseases; Privolzhsky Research Medical University, 10/1 Minin and Pozharsky Square, Nizhny Novgorod, 603005, Russia

**Keywords:** COVID-19, SARS-CoV-2, COVID-19 in adults, COVID-19 in men, Krebs index, comorbidity

## Abstract

**The aim of the study** is to assess the gender-related specifics of the COVID-19 course in patients under 55 years of age.

**Materials and Methods:**

This pilot single-center continuous retrospective non-randomized study was carried out in the repurposed infectious diseases hospital of the Privolzhsky Research Medical University (Nizhny Novgorod, Russia). The study inclusion criterion was the age of patients (up to 55 years) and confirmed coronavirus infection. In the groups based on gender differences (25 men, average age 44.0±7.8 years and 32 women, average age 41.9±9.1 years), we monitored complications of COVID-19 such as the transfer of patients to the ICU and the volume of lung damage (determined with CT scans).

**Results:**

The course of COVID-19 in male patients younger than 55 was aggravated by concomitant diseases (γ=0.36; p=0.043), among which IHD (γ=1.00; p=0.003) and liver disease (γ=0.58; p=0.007) dominated. Frequency analysis confirmed the high prevalence of coronary artery disease in men (p=0.044). Significant differences between the gender-related groups were noted in the volume of lung lesions: at admission (p=0.050), during hospital treatment (p=0.019), and at discharge (p=0.044). Using the logistic regression method, a relationship was found between the transfer of male patients to ICU and the Krebs index [y= –2.033 + 1.154 male gender + 1.539 Krebs index (χ^2^=5.68; p=0.059)] and comorbidity [y= –2.836 + 1.081 male gender + 2.052 comorbidity (χ^2^=7.03; p=0.030)]. The influence of the Krebs index and the male gender on the excess volume of lung lesions was shown [y= –1.962 + 0.575 male gender + 1.915 Krebs index (χ^2^=7.78; p=0.021)].

**Conclusion:**

In individuals under the age of 55 diagnosed with COVID-19, gender is of significant importance: in men, there is a more pronounced lesion of the lung parenchyma and a more significant change in laboratory parameters. Risk factors for a severe course of COVID-19 in men are coronary artery disease and hepatobiliary disorder. Calculating the Krebs index can be used to assess the risk of disease progression.

## Introduction

The fight against the novel coronavirus infection COVID-19 has been ongoing since December 2019, when the beta coronavirus of severe acute respiratory syndrome 2 (SARS-CoV-2) spread over the world, affecting more than 180 countries with a total of more than 191.773 million confirmed cases, of which 4.128 million were fatal [1]. The main targets of SARS-CoV-2 are pneumocytes, immune cells, and vascular endothelial cells [2]. Clinical manifestations of COVID-19 range from asymptomatic to severe forms, including life-threatening complications [3–6].

The SARS-CoV-2 virus infects people of both sexes, all ages, races, and ethnic groups [7].

Elderly people are more susceptible to SARS-CoV-2 and are more likely to be admitted to the ICU with a high risk of death [8, 9]; therefore, at present, health of patients over 60 years old are of great concern for clinicians. Age-related muscle atrophy and changes in the lung anatomy in the elderly lead to changes in the physiological function of the respiratory system [10, 11]. The progression of mitochondrial dysfunction with age leads to disorders of the immune system (e.g. impaired T cell immunity) and thus contributes to the higher susceptibility to viral infections [12, 13]. The accumulation of abnormal mitochondria may result from metabolic disorders in diabetes mellitus, oncological, neurodegenerative, and other diseases [14].

It was documented that the novel coronavirus infection caused higher mortality in men than in women [15, 16]. This might be due to the higher levels of type 1 interferon produced in the woman’s body, which is important for an early response to COVID-19 [17, 18]. The process of SARS-CoV-2 invasion and transmission is mediated by the angiotensin-converting enzyme 2 (ACE2) receptor [19]. This receptor is protected by estradiol, which is the main sex hormone in women [20–22].

In the context of the ongoing pandemic, information on risk factors of life-threatening complications is becoming highly relevant. However, the number of publications on the course of COVID-19 in patients under 55 is significantly less as compared with studies on those over 65. Information about the course of the disease in middle-aged individuals is scarce and, as a rule, limited to the description of the disease in specific regions or in certain ethnic groups [23–25].

**The aim of the study** is to assess the gender-related specifics of the COVID-19 course in patients under 55 years of age.

## Materials and Methods

This pilot single-center continuous retrospective non-randomized study was carried out in the infectious diseases hospital deployed on the basis of the University Clinic of Privolzhsky Research Medical University (Nizhny Novgorod, Russia). The work was performed in accordance with the Declaration of Helsinki (2013) and approved by the Ethics Committee of the Privolzhsky Research Medical University. All patients signed the informed consent forms to participate in the study and provide biological material.

The criterion for inclusion in the study was the patients’ age of 20 to 55 years and the confirmed coronavirus infection (according to the viral RNA amplification test). Normally, this age interval precedes the menopause period in women. The age of the group was determined in accordance with the conditional periods of biological age, based on the anatomical and physiological characteristics of the body of an adult at a mature age (20–55 years).

The study involved 57 people (mean age 42.8±8.5 years), admitted to the hospital 8 [7; 11] days after the onset of the disease. The hospitalization was due to disease exacerbation despite the preceding outpatient treatment. According to CT, the volume of pulmonary parenchyma lesions was 40 [25; 52]% upon admission and 28 [15; 48]% upon discharge. The average bed-day score was 14 [12; 17] days. Transfer to the ICU was needed for 7 patients (12.3%) aged 43.4±7.7 years, who were admitted on day 10 [8; 12] of illness with CT lesion volume of 75 [52; 80]%.

The frequency of the complicated course of COVID-19 (transfer to ICU, excess volume of lung lesions) was determined in gender-specific groups of patients. The group of men included 25 individuals (44%) aged 44.0±7.8 years with a BMI of 28.4 [26.3; 30.9], enrolled on day 8 [8; 11] of illness with an average hospital stay of 16 [12; 18] bed-days. The group of women included 32 individuals (56%) aged 41.9±9.1 years with a BMI of 27.5 [25.2; 32.0], enrolled on day 8.5 [7; 10] of illness with an average hospital stay of 13 [12; 16] bed-days. Both groups were comparable in age (p=0.49), the day of onset of the disease (p=0.81), and BMI (p=0.75). The duration of hospital stay tended to differ between men and women (p=0.096).

Comprehensive diagnosis and treatment procedures in patients with COVID-19 were carried out in accordance with the Interim Methodological Recommendations “Prevention, Diagnosis, and Treatment of Novel Coronavirus Infection (COVID-19)”, versions 6 (April 28, 2020) and 7 (June 3, 2020). These recommendations — relevant to the time of hospitalization — were approved by the Ministry of Health of Russia [26, 27] and closely corresponded to the Interim Recommendations of the World Health Organization [28].

In patients with signs of viral pneumonia, the initial assessment included a lung CT scan as the most sensitive method for detecting COVID-19-related changes. In this study, a Toshiba Aquilion 32-slice computer tomograph (Toshiba, Japan) was used.

The general (clinical) blood test (leukocytes, platelets, leukocyte formula, erythrocyte sedimentation rate (ESR)) was carried out using an XT-4000i hematology analyzer (Sysmex, Japan) and the manufacturer’s reference materials and reagents. The Krebs index was calculated as the neutrophil/lymphocyte ratio (NLR).

Biochemical blood tests with determination of creatinine, alanine and aspartate aminotransferases (ALT and AST) were performed according to Reitman–Frankel. Bilirubin, C-reactive protein (CRP) were measured using an Indiko biochemical analyzer (Thermo Fisher Scientific, Finland) and reagents from Randox Laboratories (UK). We also studied the patient’s coagulogram because intravascular coagulation is often associated with coronavirus infection. Hemostasis indices were determined by coagulometry and included activated partial thromboplastin time (APTT), prothrombin time (PTT), fibrinogen, antithrombin III (AT  III), international normalized ratio (INR), and D-dimer (quantitative method). For the study, we used an ACL TOP 500 CTS coagulometric automatic analyzer (Werfen Instrumentation Laboratory, USA); reagents were from the manufacturer, and reference materials were from RENAM (Russia).

Body mass index was calculated using the conventional Quetelet formula. The glomerular filtration rate (GFR) was estimated according to the Cockcroft–Gault formula. Comorbidity was confirmed by the presence of two or more concomitant diseases in a patient.

**Statistical analysis** of the data was carried out by methods of descriptive, parametric, and nonparametric statistics using the Statistica 10.0 software (StatSoft, USA). Laboratory and instrumental data not related to normal Gaussian distribution, are presented as a median value with border quartiles [Q1; Q3], age is indicated as a mean and standard deviation M±σ. The normality of data distribution was assessed using the Kolmogorov–Smirnov test. The significance of differences in variables other than normal distribution was assessed using the Mann–Whitney test for independent samples of quantitative indicators. The statistical relationship between the indices was produced using the Spearman (R) and Gamma (γ) nonparametric rank correlation method. Statistical testing of the absence/presence of differences in the groups was carried out according to the χ^2^ criterion. The differences between the gender groups were revealed by the frequency analysis. The dependence of the complicated course (transfer to ICU, excess volume of lung damage) on various factors (gender, Krebs index, comorbidity) was assessed by multiple logistic regression. Differences were considered statistically significant at p<0.05.

## Results and Discussion

There was only one case of death among patients studied (a 47-year-old woman); therefore, this outcome was not analyzed further.

**Figure 1:**
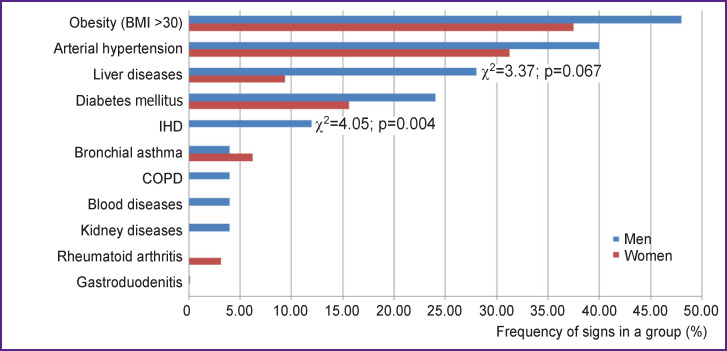
Frequency analysis of comorbidities in patients with COVID-19 under the age of 55

The majority of patients hospitalized with COVID-19 had comorbidities. Using the rank correlation method, we found that male patients under the age of 55 had two or more concomitant diseases (γ=0.36; p=0.043), among which IHD (γ=1.00; p=0.003) and liver diseases (γ=0.58; p=0.007) prevailed. Frequency analysis confirmed the prevalence of coronary artery disease in this group of men (p=0.044) as a concomitant disease (Figure 1).

Significant inter-gender differences were also noted in the extent of pulmonary parenchyma lesions as revealed by CT examination (Table 1). Upon admission to the hospital, the volume of pulmonary parenchyma lesions was higher in COVID-positive men as compared to women (p=0.050).

**Table 1:** Clinical laboratory and instrumental indicators in COVID-positive patients under 55 (Me [Q1; Q3])

Indicator	Men (n=25)	Women (n=32)	p (n=57)
CT-evidenced lung lesion volume (%):			
on admission	44 [32; 52]	32 [22; 52]	**0.050**
maximum value	48 [40; 64]	32 [22; 52]	**0.019**
at discharge	40 [28; 52]	24 [12; 40]	**0.044**
percent reduction in lung damage	22.2 [12.5; 33.3]	26.8 [15.5; 38.8]	0.350
Leukocytes (×10^9^/L):			
on admission	5.9 [5.2; 7.3]	5.3 [3.7; 7.0]	0.100
minimum value	4.4 [3.6; 5.0]	4.8 [3.7; 5.7]	0.420
at discharge	5.8 [5.0; 6.8]	5.7 [4.9; 6.6]	0.490
Lymphocytes (×10^9^/L):			
on admission	1.7 [1.1; 2.0]	1.8 [1.2; 2.2]	0.720
minimum value	1.1 [0.9; 1.6]	1.6 [0.8; 2.1]	0.160
at discharge	2.2 [1.7; 2.8]	2.3 [1.8; 2.5]	0.830
Krebs index (%):			
on admission	1.9 [1.3; 3.3]	2.0 [1.2; 2.7]	0.580
at discharge	1.3 [0.9; 1.6]	1.1 [0.9; 1.5]	0.670
Monocytes (×10^9^/L):			
on admission	1.0 [0.8; 1.4]	0.8 [0.6; 0.9]	**0.007**
maximum value	1.4 [1.1; 1.9]	0.9 [0.8; 1.1]	**0.001**
at discharge	0.8 [0.7; 1.2]	0.8 [0.6; 0.9]	**0.046**
Platelets (×10^9^/L):			
on admission	241 [169; 316]	235.5 [167.5; 301.0]	0.850
maximum value	379 [319; 491]	342.5 [282.0; 433.5]	0.150
at discharge	309 [240; 402]	282.5 [254.0; 401.0]	0.890
ESR (mm/h):			
on admission	21 [6; 38]	21.0 [11.5; 29.5]	0.770
maximum value	36 [21; 55]	22 [13; 49]	0.180
at discharge	10 [6; 21]	13.5 [9.0; 20.5]	0.610
CRP (mg/L):			
on admission	37 [22; 89]	28.5 [9.5; 101.0]	0.390
maximum value	61 [33; 142]	59.0 [14.0; 146.5]	0.590
at discharge	5 [2; 5]	4.5 [2.0; 5.0]	0.760
Total bilirubin ( μmol/L):			
on admission	12.7 [7.2; 17.6]	8.7 [5.0; 13.6]	**0.029**
maximum value	14.4 [8.6; 18.7]	16.3 [9.7; 18.1]	0.920
at discharge	9.3 [8.4; 12.7]	9.4 [5.2; 15.8]	0.450
AST (units/L):			
on admission	38 [25; 59]	28 [21; 40]	**0.043**
maximum value	68 [53; 107]	36.5 [25.5; 85.5]	**0.014**
at discharge	39 [24; 73]	28.5 [24.0; 55.5]	0.390
ALT (units/L):			
on admission	34 [28; 63]	29.0 [16.0; 41.5]	**0.043**
maximum value	107 [73; 190]	55.5 [32.0; 103.5]	**0.006**
at discharge	66 [35; 151]	38.0 [32.0; 88.5]	0.120
Creatinine (μmol/L):			
on admission	100 [91; 119]	73.5 [69.0; 84.5]	**0.001**
maximum value	120 [110; 147]	88.0 [73.0; 109.0]	**0.001**
at discharge	96 [88; 110]	73.0 [63.5; 83.5]	**0.001**
GFR (ml/min):			
on admission	119.5 [94.5; 136.8]	116.8 [100.8; 138.1]	0.960
maximum value	94.2 [86.0; 110.4]	98.5 [73.0; 116.5]	0.740
at discharge	122.3 [108.5; 148.1]	117.5 [94.4; 154.6]	0.440
D-dimer (mg/L):			
on admission	0.3 [0.3; 0.5]	0.3 [0.3; 0.4]	0.830
maximum value	0.4 [0.3; 0.9]	0.4 [0.3; 0.9]	0.590
at discharge	0.2 [0.2; 0.3]	0.3 [0.2; 0.3]	0.640
Fibrinogen (g/L):			
on admission	5.1 [4.3; 7.2]	4.5 [3.7; 5.4]	**0.045**
maximum value	6.3 [5.2; 7.2]	4.9 [3.9; 6.4]	**0.013**
at discharge	3.8 [2.8; 4.3]	3.7 [2.9; 4.3]	0.840
APTT (s):			
on admission	32.9 [29.8; 35.0]	31.0 [27.9; 33.4]	0.088
at discharge	31.8 [29.0; 34.2]	33.7 [31.1; 36.7]	0.190
PTT (s):			
on admission	12.8 [12.4; 13.7]	12.6 [11.9; 12.9]	0.076
at discharge	12.0 [11.8; 12.5]	12.0 [11.5; 12.8]	0.830
INR (units):			
on admission	1.1 [1.1; 1.2]	1.1 [1.0; 1.2]	0.170
at discharge	1.0 [1.0; 1.1]	1.1 [1.0; 1.1]	0.670
AT III activity (%):			
on admission	109 [101; 118]	106.5 [98.8; 112.0]	0.800
at discharge	102.0 [95.9; 109.0]	100.5 [92.0; 107.5]	0.830

Furthermore, the greater increase in the area of lung damage in men (as compared to women) during hospitalization (p=0.019) reflected a faster development of the infectious process; this parameter can serve as a predictor of an unfavorable course of coronavirus infection. The present result necessitates the timely initiation of therapy for coronavirus infection in men <55 years old both at the outpatient and early inpatient stage.

We also found gender-related differences in other indicators determined upon admission to the hospital: hyperbilirubinemia (p=0.029), increased hepatic transaminases AST (p=0.043) and ALT (p=0.043); those values were significantly higher in men than in women. The lab findings indicated organ dysfunction, decompensation of concomitant diseases, and the development of complications that were considered when choosing pharmacotherapy and the dosage regimen. The hepatobiliary disorders found in male patients on admission might have resulted from an unhealthy lifestyle or excessive use of medicines at the outpatient period. These disorders should be treated by the timely administration of hepatoprotectors.

The increased transaminase activity in patients with COVID-19 is thought to be a predictor of a poor outcome of the disease [29–33]. The causes of liver dysfunction in COVID-19 may vary: it can be either direct damage to hepatocytes by the SARS-CoV-2 virus or a cytokine storm result. The hepatotoxic effect of medicines used in the treatment of coronavirus infection could not be ruled out.

There was a statistically significant difference in the level of plasma creatinine between men and women throughout the entire hospital stay (p<0.001). The increase is indicative of kidney damage resulted from the cytokine-induced systemic inflammatory response. An additional mechanism might include the virus invasion mediated by the angiotensin-converting enzyme 2 receptor expressed in the kidney [34]. It is known that patients with kidney disease have a significantly higher risk of death in hospital; independently, a high level of serum creatinine is a risk factor for hospital mortality [35].

A relationship was found between the decrease in the absolute value of lymphocytes and the severity of the course of COVID-19 [36]. We have shown the absence of statistically significant differences in the level of leukocytes and lymphocytes in gender groups, the indicators are reduced in both men and women. Abnormally high presence of monocytes in male patients was noted at admission (p=0.007), at discharge (p=0.046), and during treatment in the hospital (p<0.001). Monocytosis is usually associated with severe disease. We found that the increase in the level of monocytes directly correlated with the male gender (γ=0.50, p=0.001; R=0.82, p=0.001) and the volume of pulmonary parenchyma lesions (γ=0.39, p=0.003; R=0.32, p=0.014). The increase in the number of monocytes correlated with the severity of the disease course, namely with the transfer to the ICU (γ=1.00, p=0.001; R=0.33, p=0.011) and the initiation of biological (γ=0.56; p=0.003) and hormonal (γ=0.56; p=0.002) therapy.

There is ambiguous information about the effect of monocytosis on the dynamics of coronavirus infection. Thus, Hensel et al. [37] demonstrated a relationship between the cardiological history and mortality of patients with monocytosis (OR=3.91 [1.87; 8.18]; p<0.001); patients with respiratory symptoms (p<0.001) and infection (p<0.001) sought medical attention more often. The statistically significant increase in the monocyte count in COVID-positive men corroborates with the well-known correlation of this indicator with the disease severity.

There were no significant differences in ESR and CRP values between men and women. The high level of acute-phase proteins in both groups was used to monitor the inflammatory process and prescribe appropriate antibacterial, biological, and hormonal therapy [38].

An increased level of D-dimer and fibrinogen on the backdrop of normal PTT and platelet count at the initial stage of the disease is characteristic of the COVID-associated coagulopathy in contrast to coagulopathies caused by bacterial sepsis or disseminated intravascular coagulation [39, 40]. It is, therefore, seen as a poor prognostic factor for developing thrombotic complications [41–45].

Despite the increase in the amount of D-dimer and fibrinogen in most of the patients, a significant difference between the gender groups was found only for fibrinogen at admission (p=0.045) and during treatment (p=0.013). Fibrinogen is a precursor of fibrin and an acute-phase protein. Increased levels of fibrinogen could reflect the stimulation of its biosynthesis in the process of microthrombi formation or, conversely, its suppressed catabolism in the lungs [46, 47].

The values of the chronometric parameters (APTT, PTT) as well as the activity of AT III as criteria of the anticoagulant potential in our patients did not exceed the reference limits. There was a trend towards gender-related differences in the APTT (p=0.088) and PTT (p=0.076), which might be caused by variations of the liver function [48, 49].

The mechanism of coagulopathy in COVID-19 is not fully understood. The one could involve dysregulated immune responses caused by inflammatory cytokines, lymphocyte death, hypoxia, or endothelial damage [50]. On the one hand, an increased thrombus formation may limit the spread of the SARS-CoV-2 virus, on the other hand, endothelial damage inhibits thromboprotection and allows for excessive thrombin production, dysregulation of fibrinolysis, and thrombus formation [5, 51–53].

We considered that the increased values of laboratory indices (monocytes, hepatic transaminases, bilirubin, creatinine, and fibrinogen) might be mainly due to concomitant diseases. The aggravated course and progression of the viral infection in comorbid COVID-positive patients has been documented [54–60]. The duration of a hospital stay and the number of adverse outcomes were higher in patients having two or more comorbidities [61]. It is, therefore, necessary to monitor laboratory parameters reflecting the excretory body functions in order to initiate an adequate early therapy. To reduce the risk of toxic liver damage, the use of hepatoprotective and choleretic agents in patients with COVID-19 may be justified and should be further discussed.

**Figure 2:**
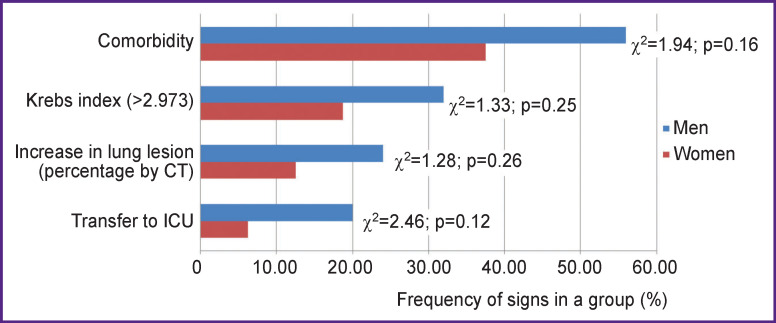
Frequency analysis of the complicated course of COVID-19 and factors of its development in patients under 55

In this study, we also assessed the role of comorbidity and the Krebs index (higher than 2.973) as factors affecting the course of COVID-19 in patients under 55 years of age. The event of patient transfer to the ICU and the increase in the lung damage area were used as markers of the complicated course of the disease. The risks and occurrences of these two events for men and women are shown in Figure 2. There were some differences between the two genders but they did not reach statistical significance.

The present results suggest that gender is an essential factor affecting the course of coronavirus infection in adult patients under 55 years old. A meta-analysis [62] identified male sex as a risk factor for SARS-CoV-2 infection, transfer to ICU (OR=2.84 [2.06; 3.92]; p<0.001) and likelihood of death (OR=1.39 [1.31; 1.47]; p<0.001). Using the method of multiple logistic regression, we revealed a relationship between the complicated course of COVID-19 and the male gender in combination with the Krebs index and comorbidity (Table 2). The male gender, as well as comorbidity, increased the likelihood of transfer to the ICU in COVID-19 patients (χ^2^=7.03; p=0.030). It should be noted that both the male gender and the Krebs index value (χ^2^=5.68; p=0.059) contributed almost equally to the frequency of transfer to the ICU.

**Table 2:** Relationship between gender and indicators of the complicated course of COVID-19 in patients under 55

Indicator	Regression equation
Transfer to ICU	y= –2.836 + 1.081 male gender + 2.052 comorbidity (χ^2^=7.03; p=0.030)
	y= –2.033 + 1.154 male gender + 1.539 Krebs index (χ^2^=5.68; p=0.059)
Increased percentage of lung damage	y= –1.962 + 0.575 male gender + 1.915 Krebs index (χ^2^=7.78; p=0.021)
	y= –0.913 + 0.881 male gender + 0.451 comorbidity (χ^2^=1.66; p=0.436)

During the period of hospital treatment, an increase in the lung damage area was noted in 10 patients (17.5%), including 4 women and 6 men; the multivariate analysis showed a correlation between the lung damage and the Krebs index as well as the male gender (χ^2^=7.78; p=0.021).

There was no statistically significant effect of comorbidity on the increase in the volume of lung damage in male patients under study (χ^2^=1.66; p=0.436).

There were no differences in the Krebs index between the gender groups. Its value on admission of COVID-positive patients was used as a prognostic indicator of the disease severity. Thus, NLR ≥2.973 is regarded as a risk factor for disease progression during hospitalization [63], and with NLR >6.11, there is a high risk of death [64]. Therefore, calculating the Krebs index in COVID-positive male patients with comorbidity can be used to assess the risk of disease progression at an earlier stage. To predict the course of the disease it is advisable to introduce the determination of the Krebs index into the standards of patient care in COVID-19.

### Limitations of the study

This study has some limitations. The presence/absence of concomitant diseases were based on patient’s history either documented or reported verbally. Therefore, we cannot rule out overdiagnosis in these reports. At the same time, we were able to confirm the preliminary diagnoses when observing the patients during hospitalization. No population conclusions can be drawn from these data due to the limited number of patients under study.

## Conclusion

The course of COVID-19 in men and women under 55 years depends on patient’s gender: men have more pronounced damage to the lung parenchyma and a more significant change in laboratory parameters. Risk factors for a severe course of COVID-19 in men are coronary artery disease and disorders of the hepatobiliary system. Calculating the Krebs index can be used to assess the risk of disease progression.
